# The importance of socio-economic context for social marketing models for improving reproductive health: Evidence from 555 years of program experience

**DOI:** 10.1186/1471-2458-5-10

**Published:** 2005-01-27

**Authors:** Dominique Meekers, Stephen Rahaim

**Affiliations:** 1 Department of International Health and Development, School of Public Health and Tropical Medicine, Tulane University, 1440 Canal Street, Suite 2200, New Orleans, LA 70112, USA; 2 Chemonics International, Washington, D.C., USA

## Abstract

**Background:**

Over the past two decades, social marketing programs have become an important element of the national family planning and HIV prevention strategy in several developing countries. As yet, there has not been any comprehensive empirical assessment to determine which of several social marketing models is most effective for a given socio-economic context. Such an assessment is urgently needed to inform the design of future social marketing programs, and to avoid that programs are designed using an ineffective model.

**Methods:**

This study addresses this issue using a database of annual statistics about reproductive health oriented social marketing programs in over 70 countries. In total, the database covers 555 years of program experience with social marketing programs that distribute and promote the use of oral contraceptives and condoms. Specifically, our analysis assesses to what extent the model used by different reproductive health social marketing programs has varied across different socio-economic contexts. We then use random effects regression to test in which socio-economic context each of the models is most successful at increasing use of socially marketed oral contraceptives and condoms.

**Results:**

The results show that there has been a tendency to design reproductive health social marketing program with a management structure that matches the local context. However, the evidence also shows that this has not always been the case. While socio-economic context clearly influences the effectiveness of some of the social marketing models, program maturity and the size of the target population appear equally important.

**Conclusions:**

To maximize the effectiveness of future social marketing programs, it is essential that more effort is devoted to ensuring that such programs are designed using the model or approach that is most suitable for the local context.

## Background

In many developing countries, social marketing program have become an essential component, if not the main component, of the national family planning and HIV prevention strategy. Social marketing programs in reproductive health all aim to improve reproductive health of the target population by using commercial approaches to promote healthy behaviors and/or to expand access to essential products and services. While improving reproductive health of the target population is the ultimate aim, donors recognize the potential of social marketing programs to build the sustainable delivery of reproductive health and family planning products and services in developing countries. Different types of social marketing models have been used to achieve these objectives. Social marketing programs have often been classified as either using the *non-governmental organization (NGO) model (*sometimes also referred to as the *social marketing organization model*) or the *manufacturer's model *. [1-3]. The NGO model typically focuses on achieving the largest possible health impact among the target population. Hence, the NGO model typically heavily subsidizes products. By contrast, the manufacturer's model was specifically developed in response to the need not only to improve reproductive health, but also to do so in a financially sustainable manner. Programs under the manufacturer's model are treated as temporary interventions with a realistic exit strategy. Such programs typically use experts to provide temporary technical assistance to existing private sector companies, which eventually are expected to continue the program without subsidies. Although the two models are defined largely by the type of management and financial structure, they often also differ in terms of branding, pricing, distribution, and other factors. Moreover, context-specific variations on these two models are common, and some programs use hybrid approaches that integrate features from each of the two broader models [4].

While many social marketing variants have proven successful, it is generally assumed that the manufacturer's model is more feasible in middle-income countries with a fairly well developed commercial infrastructure, while NGO models are more appropriate in lower-income countries with less less-developed commercial infrastructure [4]. Although scattered case studies support these assumptions, as yet there has not been any comprehensive empirical assessment of the socio-economic context in which each model works best.

This paper analyzes a comprehensive database of annual social marketing statistics. The database is unique in that it includes annual data on social marketing programs in reproductive health in over 70 countries, which cumulatively represent 555 years of experience with social marketing programs. We analyze these data to provide empirical information on the extent to which different social marketing models have been used in different socio-economic contexts, and to identify the socio-economic context in which each model is most effective. Our analysis is restricted to programs that social market condoms or oral contraceptives. As only limited data on the features of social marketing programs are routinely collected, we focus on differences in management structure.

In many countries, social marketing programs are an essential component of the national family planning and HIV prevention programs. At present, large-scale social marketing programs for reproductive health are in operation in over 60 countries worldwide. A growing body of evidence shows that social marketing programs can make an important contribution to the reproductive health of the target population [5-19].

In 2002, social marketing programs in 69 countries sold almost 1.6 billion condoms [20] accounting for more than half of all condoms available from public sources. Total sales of condoms, pills, vaginal foaming tablets and IUDs amounted to over 28 million couple-years of protection (a measure of the number of couples that could be protected from pregnancy for one year). In these countries (excluding China and Russia) social marketing sales of temporary methods of contraception excluding condoms accounted for almost 10 percent of all use [21].

Social marketing tends to be a cost-effective approach to achieving widespread access to health products and services [22,23]. It uses private sector incentives and organizations to achieve efficiency and revenues from sales offset some of the program costs. The total cost of $6 per couple-year of protection compares favorably with costs to implement public sector family planning programs, which are around $15–20 per couple-year of protection [24-27]. Some programs may eventually become self-sustaining and graduate from donor assistance altogether. Some social marketing programs in low-income countries are now operated by independent local social marketing foundations (e.g., the Ghana Social Marketing Foundation (GSMF) and ADEMAS in Senegal) and in some middle-income countries social marketing sales are continuing well after donor assistance has ended (e.g., condoms in Turkey, oral contraceptives in Morocco, and injectables in Brazil) [28-30].

Three different management structures are common in social marketing programs: 1) management by an affiliate of an international NGO, 2) management by local clinic-based or non-clinic-based organizations, and 3) partnerships with a commercial organization. The most commonly used management structure in health-oriented social marketing programs is management by an NGO. Typically this implies management by an affiliate of an international social marketing organization that provides an expatriate resident advisor. Several social marketing programs are managed by local organizations. For example, some social marketing programs are managed by a specially developed new organization that is independent of international affiliation. These are often called *social marketing foundations*. This organization may receive technical assistance from an international social marketing organization but it operates as an independent entity. Implementation may also be by an existing organization, such as a family planning association (FPA) or another type of clinic-based organization. In such case an international NGO provides technical assistance to the local organization to develop and operate the social marketing program. Social marketing programs may also be implemented through a partnership between an international NGO and an existing commercial organization. In this case, the international NGO often helps to develop the market for the product through research and promotion, while the commercial partner handles all aspects of packaging, sales and distribution.

Not all management approaches are feasible in every context. For example, in countries with relatively high-income levels and a strong commercial sector there may be many opportunities for commercial partnerships, while in other countries there may be few such opportunities, if any [4]. Similarly, opportunities for management through existing local organizations do not exist in all countries. Furthermore, the context in which social marketing programs are implemented has a crucial impact on the types of programs that can be successful. For example, in a poor country with a rudimentary commercial sector, it is unlikely that commercial partnerships will be successful. On the other hand, in a middle-income country with a well-developed commercial sector and a rapidly developing middle class, it may be counterproductive to use an NGO affiliate model with a highly subsidized product that competes with existing brands.

## Methods

This section analyses empirical data to study differences in social marketing models and their impact. Since data about program characteristics are scarce, we focus on differences in management structure. Specifically, our objectives are to 1) describe to what extent the type of social marketing management structure actually varies according to the socio-economic context in which the programs are implemented, and 2) to examine the effectiveness of various management structures in increasing socially marketed product sales in different contexts.

### Data

For the purpose of this study, we compiled a database that comprises data on most of the major social marketing programs that were in operation at any time during the period between 1988 and 2001. Many, but not all, of these programs were still in operation in 2001. The database contains annual data on several program characteristics and on indicators of the local socio-economic context. The main sources of information for data on program characteristics were DKT International's "Contraceptive Social Marketing Statistics," Population Services International's (PSI) MIS database, and published data on Futures Group International (TFGI) programs [26]. These three programs are the dominant implementing organization in contraceptive social marketing [1]. Data on the local context were obtained from the "World Development Indicators 2003 CD-ROM" [31].

For each social marketing program, the database contains one case for each year of operation within the 1988–2001 study period. In other words, our unit of analysis is the "program-year." Using "program-years" as our unit of analysis has the advantage that all indicators, including program characteristics, can change over time. In addition, it implies that in the analyses programs that have been in operation for a longer period of time are given more weight than newer programs. For example, a social marketing program that has been operating since 1988 will contribute 14 program-years to the database (one case for each year of operation); a program that started in 1995 and was terminated in 1997 will contribute three program-years, and a program that started in 2001 will contribute only one program-year.

After restricting the database to those social marketing programs that have a reproductive health component, the database contains information on social marketing programs in over 70 countries, which represent a total of 555 years of program experience. This includes 508 years of experience with condom social marketing and 208 years of experience with OC social marketing.

#### Indicators

Our measures of market potential are based on the indicators used by Michigan State University's Center for International Business Education and Research [32]. Specifically, we use the following indicators of market size, market intensity, and commercial infrastructure:

#### Market size

• Population size

• Percentage of population living in urban areas

#### Market intensity

• Per capita GNI in current international dollars. This indicator measures the GNI converted in international dollars using purchasing power parity. An international dollar has the same purchasing power as a dollar in the U.S. [World Bank 2003]

#### Commercial infrastructure

• Telephone mainlines per 1,000 inhabitants

• Television sets per 1,000 inhabitants

The database also includes selected indicators of the characteristics of the social marketing programs, including:

• Management structure (NGO affiliate, local clinic-based and non-clinic-based organizations, and commercial partnerships)

• Program maturity/years of operation (less than 3 years, 4–6 years, and 7+ years)

• Number of reproductive health products social marketed (one vs. two or more). Only condoms, oral contraceptives, injectables and IUDs are considered.

In addition to these indicators of the socio-economic context and of the characteristics of the programs, information is available on the following outcome measures:

• Per capita condom sales

• Per capita OC sales

While some social marketing programs distribute and/or promote injectables or IUDs, their number is not sufficiently large to allow multivariate analyses. Hence, data on sales of injectables and IUDs are not examined in this study. Other indicators of program effectiveness, such as user-prevalence rates are not collected on an annual basis.

Ideally, one would also want to examine the cost-efficiency of implementing different social marketing models. A commonly used approach to estimate cost-efficiency is to conduct a cost-effectiveness analysis. By definition, any cost-effectiveness analysis requires obtaining data on both the effectiveness of the program, i.e. the impact of the program, and on the cost of implementing the program [33,34]. Unfortunately, only limited cost information on social marketing programs is available, and the cost data that are routinely collected by the main social marketing organizations are not comparable. Hence, we are unable to assess the cost-efficiency of the programs in our database.

### Methods

In the next sections, we use cross-tabulations to examine to what extent management structure varies by socio-economic context. Since our unit of analysis consists of program-years of operation, these tabulations show the percentage of program-years that had each management type.

We also test the extent to which different indicators of socio-economic context affect effectiveness of social marketing programs with different management structures. For this purpose, we use multiple regression analyses to assess the effect of indicators of socio-economic context on per capita condom sales for different management structures. Because each social marketing program has multiple entries in the data set (one for each year of operation), the standard errors in ordinary linear regression are incorrect. We use random-effects regression to solve the problem that observations for a given country-program are not independent [35].

For each type of management structure, we assess the net effect of socio-economic context on per capita condom sales, after controlling for the number of reproductive health products marketed, program maturity, time period (1995–2001 vs. earlier). Similar analyses are conducted for per capita OC sales.

## Results

### Variations in management structure by socio-economic context

We now analyze our database to illustrate to what extent different management structures have been used in social marketing programs (see Table 1). Overall, data on the management structure of the social marketing programs were available for 555 program years. Of these 55 program years, 72% were managed through an NGO affiliate, 16% by local organizations, and 12% through a commercial partnership.

**Table 1:** Distribution of Years of Social Marketing Program Experience by Management Structure and by Socio-Economic Context

	Distribution of Program Years			
		
	% NGO Affiliate	% Local Organizations	% Commercial Partnership	No. of Program Years
Population Size				
<10 Million	76.3%	17.1%	6.6%	211
10–25 Million	62.7%	24.0%	13.3%	150
25+ Million	72.5%	9.0%	18.5%	189
				
Urban Population				
<25%	86.1%	13.9%	0.0%	101
25–49%	80.8%	7.7%	11.5%	260
50+%	47.0%	31.0%	22.0%	168
				
GNI per capita				
<$1000	97.0%	3.0%	0.0%	132
$1000–3000	68.2%	18.6%	13.2%	258
$3000+	52.3%	24.5%	23.2%	151
				
Phone mainlines per 1,000				
<5	87.1%	11.8%	1.1%	178
5–30	73.4%	9.2%	17.4%	184
30+	53.8%	27.4%	18.8%	186
				
Television sets per 1,000				
<20	88.2%	10.1%	1.8%	169
20–100	70.9%	18.7%	10.4%	182
100+	48.5%	23.3%	28.2%	163
				
Time Period				
1986–93	56.4%	25.4%	18.3%	126
1994–97	66.2%	18.5%	15.3%	216
1998–01	85.9%	8.0%	6.1%	213
				
Region				
Eastern Europe	100.0%	0.0%	0.0%	16
Africa	89.7%	7.7%	2.6%	273
Asia	64.2%	17.5%	18.3%	137
Latin America	42.9%	41.9%	15.2%	105
Mid. East/N. Afr.	13.6%	0.0%	86.4%	22
				

Total	71.5%	16.0%	12.4%	555

Social marketing programs in countries with populations smaller than 10 million have predominantly been managed by NGO affiliates (76% of program-years). In larger countries, other management structures have been somewhat more common.

Breakdown by level of urbanization confirms that the range of management strategies used increased with socio-economic status. The percentage of social marketing program-years managed by NGO affiliates ranged from 86% when urbanization was low to 47% when urbanization was high. In highly urbanized populations, 31% of program-years were managed by existing local organizations and 22% by commercial partnerships.

Social marketing programs in countries with a low per capita GNI (<$1,000) have been managed predominantly by NGO affiliates (97% of program-years), with local organizations being a distant second (3%). In countries with higher GNI levels, a wider range of management strategies has been used. When the per capita GNI levels are medium-low, management through NGO affiliates was still the most common (68% of all program-years). However, in medium-low GNI countries local organizations accounted for 18% of program years and commercial partnerships for 13%. Finally, when the GNI exceeded $3,000 per capita, NGO affiliates accounted for only 52% of program-years, followed by local organizations (25%) and commercial partnerships (23%).

Our indicators of the level of development of the commercial infrastructure show that the NGO model has dominated in settings with a relatively poorly developed commercial infrastructure. For example, NGO affiliates accounted for roughly 87% of program years in countries with fewer than 5 phone mainlines per 1,000 inhabitants, and for 88% of program years in countries with fewer than 20 television sets per 1,000 in habitants. Management through existing local organizations and commercial partnerships is most common in settings with over 30 phone mainlines and over 100 television sets per 1,000 inhabitants.

Management structures have also changed over time. For example, the percentage of program-years managed through NGO affiliates has steadily increased from 56% in 1986–93 to 86% in 1998–2001. Simultaneously, management through existing local organizations and commercial partnerships has gradually declined.

The last panel of Table 1 shows that management through commercial partnerships has been most common in the Middle East and Northern Africa (87% of program-years), while management through existing organizations has been most common in Latin America (86% of program-years). In Eastern Europe and Africa management through NGO affiliates has dominated (90% and over). In Latin America, management through NGO affiliates and through local organizations have both been common (43% and 42%, respectively).

### The effect of socio-economic context on the effectiveness of different social marketing management structures

We now use random-effect GLS regression analyses to examine to what extent market size, market intensity, commercial infrastructure, and program features (number of products social marketed and program maturity) affect per capita condom sales and per capita OC sales. The variable "number of television sets per 1,000 inhabitants" was removed from the multivariate analyses as it correlated too much with some of the other predictor variables. We conduct separate analyses for social marketing programs managed by NGO affiliates, commercial partnerships, and local organizations. Due to the small number of cases, we are unable to differentiate between existing organizations and new organizations.

#### Management by NGO affiliates

The second column in Table 2 shows the predictors of per capita condoms sales in social marketing programs managed by NGO affiliates. The results indicate that among social marketing programs managed through NGO affiliates per capita condoms sales increase significantly with the number of years of program maturity (β = .181 for programs with 4–6 years of maturity; β = .348 for programs with 7 or more years of maturity). Programs that social marketed two or more products have higher per capita condom sales than programs that just market condoms (β = .159).

**Table 2:** Effect of Socio-Economic Context on Condom Sales and CYP Among Social Marketing Programs Managed Through an NGO Affiliate (Random-Effects Regression GLS Coefficients)

	Per Capita Condom Sales	OC Sales
		
Markets Two or More Products	.159***	.017
		
Program Maturity		
1–3 (ref)		
4–6	.181***	.001
7+	.348***	.003
		
Time Period		
<1994 (ref)		
1995–2001	.143**	.004
		
Population (in 10 millions)	-.006***	-.000*
		
Percent Urban	-.002	-.000
		
Per Capita Gross National Income	-.003	.007
		
Phone mainlines per 1,000	-.001	-.000
		
Constant	.260***	(dropped)
		
R Square	.236	.082
Number of Program-Years	374	90
Number of Countries Included	58	26

*** p < .01; ** p < .05; * p < .10

As expected, social marketing programs managed through an NGO affiliate tend to be more effective, as measured by per capita condom sales, in countries with smaller populations (β = -.006). Per capita condom sales do not vary with the level of urbanization, per capita Gross National Income (an indicator of market intensity), nor with the number of phone mainlines (an indicator of the strength of commercial infrastructure).

The third column in Table 2 shows the predictors of per capita OC sales. The results indicate that there is no evidence that our indicators of market size, market intensity, and commercial infrastructure have any significant impact on per capita OC sales.

#### Management by commercial partnerships

Table 3 shows the predictors of per capita condom sales in social marketing programs managed using the manufacturer's model. The results shown in Table 3 indicate that among social marketing programs managed through commercial partnerships, per capita condom sales tend to be higher in countries with smaller populations (β = -.012), and with a lower per capita gross national income (β = -.111). The level of commercial infrastructure, as measured by the number of telephone mainlines per 1,000 inhabitants, has a small but significant positive effect on per capita condom sales (β = .001).

**Table 3:** Effect of Socio-Economic Context on Condom Sales and CYP Among Social Marketing Programs Managed Through the Manufacturer's Model (Random-Effects Regression GLS Coefficients)

	Per Capita Condom Sales	OC Sales
Markets Two or More Products	.078	-.015*
		
Program Maturity		
1–3 (ref)		
4–6	-.018	.003
7+	-.069	.007
		
Time Period		
<1994 (ref)		
1995–2001	.099	.007
		
Population (in 10 millions)	-.012***	-.002
		
Percent Urban	.001	-.000
		
Per Capita Gross National Income	-.111**	.015**
		
Phone mainlines per 1,000	.001**	-.000
		
Constant	.278**	.030
		
R Square	.384	.185
Number of Program-Years	54	55
Number of Countries Included	9	12

*** p < .01; ** p < .05; * p < .10

The third column in Table 3 reveals a different pattern for factors affecting per capita OC sales. Among social marketing programs managed through commercial partnerships, per capita OC sales appear to be lower for those programs that also market other products (β = -.015; p < .010). Our indicator of market intensity, per capita gross national income, has a significant positive effect on per capita OC sales (β = .015). However, population size and urbanization do not have any significant effect.

#### Management by local clinic-based and non-clinic-based organizations

Table 4 shows similar analyses for programs managed by local organizations (including both clinic-based and non-clinic-based organizations). The results show that programs in countries with larger populations have significantly higher per capita condom sales (β = .036). However, program in countries with higher levels of urbanization have lower per capita condom sales (β = -.012).

**Table 4:** Effect of Socio-Economic Context on Condom Sales and CYP Among Social Marketing Programs Managed Through Local Organizations (Random-Effects Regression GLS Coefficients)

	Per Capita Condom Sales	OC Sales
		
Markets Two or More Products	.075	-.012
		
Program Maturity		
1–3 (ref)		
4–6	.158	.022*
7+	.181	.028**
		
Time Period		
<1994 (ref)		
1995–2002	.138	-.010
		
Population (in millions)	.036**	.008**
		
Percent Urban	-.012***	.001
		
Per Capita Gross National Income	.006	.028***
		
Phone mainlines per 1,000	.001	-.001***
		
Constant	.436**	-.021
R Square	.309	.401
Number of Program-Years	80	63
Number of Countries Included	17	12

*** p < .01; ** p < .05; * p < .10

The third column in Table 4 shows that among social marketing programs implemented by local organization, OC sales are significantly higher for those programs that have been in operation for seven or more years (β = .031). For these types of social marketing programs per capita OC sales increase with population size (β = .008) and with level of urbanization (β = .028). The level of commercial infrastructure, as measure through the number of telephone mainlines per 1,000 inhabitants, has a small negative effect on per capita OC sales.

## Discussion

Several studies have commented on the differences between social marketing programs that use the non-governmental organization model and those that use the manufacturer's model [1-3]. Although it is increasingly recognized that context-specific variations on these models are common, and that hybrid models exist [4], it is generally assumed that social marketing programs that use the manufacturer's model are most feasible in middle-income countries with a fairly well-developed commercial infrastructure, while NGO managed models work best in lower-income countries with less-developed commercial infrastructure. As yet, however, there has been little empirical evidence to verify these assumptions.

This paper has used a database of annual data on social marketing programs in over 70 countries to verify the extent to which the different social marketing models that have been used vary across socio-economic contexts, and to test in which socio-economic context each of the models is most successful at increasing use of socially marketed products.

A rigorous analysis of the relative effectiveness of different social marketing models would require detailed data about the characteristics of the program, including data about financial structures, branding, pricing (and level of subsidies), distribution, and promotion.

Because data on the features of social marketing programs are limited, we focused on differences in the programs' management structure. Ideally, we would also like to examine are range of outcome measures, including per capita sales, the user-prevalence, and the unit-cost for each method. Since such prevalence and unit-cost data are unavailable, we have focused on per capita condom and OC sales.

We have used empirical analyses to obtain a better understanding of the variations that exist in social marketing programs. Specifically, we have analyzed a database of annual statistics on social marketing programs in over 70 countries, accounting for a total of 555 years of program experience. The database contains annual data on the management structure, program maturity, the number of reproductive health products marketed, and on product sales. The database also contains indicators of the local socio-economic context (market size, market intensity, and commercial infrastructure), including the population size, level of urbanization, per capita GNI, number of telephone mainlines and television sets per 1,000 inhabitants.

Our analyses have shown that of the 555 years of social marketing program experience covered by the database, over 70% has used the NGO management structure. Nevertheless, there are considerable variations in management structure. The NGO affiliate management structure has been the dominant model when the per capita GNI is below $3,000 and the level of urbanization below 50%. The data also show that use of the NGO management structure has increase dramatically over the course of our study period.

The findings support the theory that there may be more opportunities for successful partnerships with the private sector in countries with a high per capita GNI. The highest percentage of such partnerships (23%) was found in countries with a GNI exceeding $3,000 per capita. This percentage decreases to 13% in countries with a per capita GNI between $1,000 and $3,000. None of the commercial partnerships in our database were in countries with a per capita GNI below $1,000. One of the reasons for this pattern may be that there is more commercial activity and a wider range of possible partners in countries with a higher per capita income. Users in those countries are also more likely to be able to afford the commercially sustainable brands that are marketed under this approach.

Although the program characteristics usually appear to be appropriate for the local socio-economic context, it is questionable whether this has always been the case. For example, over 50% of program-years in countries with a medium-high GNI (over $3,000) used the NGO model, even though countries with such income levels typically provide opportunities for commercial partnerships.

Our multivariate analyses identified the factors that affect per capita condom and OC sales for each of the four social marketing models. The reader is cautioned, however, that condom and OC sales may also be influenced by other factors that were not measured in our data base, such as HIV prevalence and overall levels of contraceptive prevalence. Bearing these cautions in mind, the analyses yielded the following key findings:

### Programs managed by NGO affiliates

• Per capita condom sales are higher in countries with smaller populations. This may be influenced by the difficulty of achieving high distribution coverage in large, mostly rural countries (e.g., India, Indonesia, Vietnam, Nigeria) and/or the time it takes to large such large populations with social marketing campaigns. Also, social marketing programs in countries with a larger population may have more competition (e.g., India, South Africa). By contrast, several small countries have a high HIV/AIDS prevalence and/or limited method choice (e.g., sub-Saharan Africa);

• Per capita condom sales are higher for more mature programs and those that market more than one product. This probably reflects improvements in distribution capability over time, as well as the cumulative effect of behavior change programs that stimulate demand. More developed programs with stronger distribution are also more likely to market several products;

• Per capita condom sales do not vary with the level of urbanization, per capita income, or commercial infrastructure. These variables are more likely to affect access-related factors, such as product access and ability to pay. Other factors to look for that may affect sales are related to demand: level of effort of the social marketing program (which reflects funding levels), health context (HIV/AIDS and contraceptive prevalence) and competition from the public sector and other NGOs that may cancel out the higher market potential in the more developed countries;

• None of the factors examined had any significant influence on the per capita OC sales of social marketing programs managed through an NGO affiliate.

### Programs managed by commercial partnerships

• Per capita condom sales tend to be higher in countries with smaller populations and with a lower per capita growth. At first glance, this finding is counter-intuitive. That is, initially one would expect sales for commercial partnerships to increase with population and per capita GNI, because commercial program need both a high demand and ability to pay. However, as population size and income increase sales may decline due to increased competition from commercial and other suppliers. Several countries with middle-high incomes (Turkey, the Philippines, and Jordan) or large populations (Turkey, the Philippines, and Indonesia) have strong public sector programs that offer free contraceptives, as well as many commercial suppliers. Also, demand for condoms in these countries has been very low, given that HIV prevalence is low that other contraceptive methods are available. By contrast, smaller countries such as Haiti and Zimbabwe have high HIV rates, less choice of methods, and probably less competition from other suppliers.

### Programs managed by local clinic-based and non clinic-based organizations

• Programs in countries with larger populations have significantly higher per capita condom sales. One likely explanation for this pattern is that local organizations tend to have good distribution networks, which pays off in countries with a larger population. Some of the larger countries (e.g., Bangladesh and Colombia) have well-established organizations with a big market share. For example, in Bangladesh SMC provides almost all condoms and the demand is very high;

• OC sales are significantly higher for programs that have been in operation for seven or more years. The longer a program operates, the more developed its distribution is likely to be. Social marketing communication efforts are also more likely to pay off over the long run;

• Programs in countries with a higher level of urbanization have lower per capita condom sales. This was anticipated, given that NGOs and family planning associations have a strong track record of serving users in peri-urban and rural areas. In addition, when urbanization increases, access to commercial products sold in pharmacies and other private sector outlets increases as well, which allows commercial suppliers to grow their market share and compete with social marketing and public sector products;

• The level of commercial infrastructure, as measured through the number of telephone mainlines per 1,000 inhabitants has a small negative effect on per capita OC sales. This finding may reflect that a better commercial infrastructure implies more competition from commercial suppliers.

## Conclusions

Our analyses of records on 555 years of experience with social marketing programs have illustrated the tendency to design social marketing programs with a management structure suitable for the local context. However, the evidence also shows examples where this has not been the case. While socio-economic context clearly influences the effectiveness of some of the social marketing models, program maturity and the size of the target population appear equally important. Consequently, it is crucial that social marketing programs are designed using the model or approach that is most suitable for the local context.

## Competing interests

Dr. Meekers is the former Research Director of PSI (1996–2001), and has been a paid consultant for other PSI research projects.

## Author's contributions

Dr. Meekers developed the study design, assisted with the analysis, and contributed to the writing of the final manuscript. Mr. Rahaim collected data, conducted literature reviews, and contributed to the editing of the paper.

## Pre-publication history

The pre-publication history for this paper can be accessed here:


